# Chitosan Nanoparticles at the Biological Interface: Implications for Drug Delivery

**DOI:** 10.3390/pharmaceutics13101686

**Published:** 2021-10-14

**Authors:** Noorjahan Aibani, Raj Rai, Parth Patel, Grace Cuddihy, Ellen K. Wasan

**Affiliations:** College of Pharmacy and Nutrition, University of Saskatchewan, 107 Wiggins Rd, Saskatoon, SK S7N 5E5, Canada; nia986@usask.ca (N.A.); raj.rai@usask.ca (R.R.); parthpatel_11@ymail.com (P.P.); cac579@mail.usask.ca (G.C.)

**Keywords:** chitosan nanoparticles, biological, cellular uptake, intracellular, biodistribution

## Abstract

The unique properties of chitosan make it a useful choice for various nanoparticulate drug delivery applications. Although chitosan is biocompatible and enables cellular uptake, its interactions at cellular and systemic levels need to be studied in more depth. This review focuses on the various physical and chemical properties of chitosan that affect its performance in biological systems. We aim to analyze recent research studying interactions of chitosan nanoparticles (NPs) upon their cellular uptake and their journey through the various compartments of the cell. The positive charge of chitosan enables it to efficiently attach to cells, increasing the probability of cellular uptake. Chitosan NPs are taken up by cells via different pathways and escape endosomal degradation due to the proton sponge effect. Furthermore, we have reviewed the interaction of chitosan NPs upon in vivo administration. Chitosan NPs are immediately surrounded by a serum protein corona in systemic circulation upon intravenous administration, and their biodistribution is mainly to the liver and spleen indicating RES uptake. However, the evasion of RES system as well as the targeting ability and bioavailability of chitosan NPs can be improved by utilizing specific routes of administration and covalent modifications of surface properties. Ongoing clinical trials of chitosan formulations for therapeutic applications are paving the way for the introduction of chitosan into the pharmaceutical market and for their toxicological evaluation. Chitosan provides specific biophysical properties for effective and tunable cellular uptake and systemic delivery for a wide range of applications.

## 1. Introduction

The nontoxic, biocompatible, and biodegradable properties of chitosan make it a polymer of choice for many biomedical and pharmaceutical applications. Chitosan microspheres and NPs for drug delivery were first reported in the late 1990s [1,2,3]. The chemical versatility of chitosan relies on its ability to form a poly-cationic charged molecule at physiological pH due to the protonation of D-glucosamine in its polymeric structure and its modifiable molecular weight [4]. Chitosan is a product of the deacetylation of chitin, and its chemical and biological properties are dependent on the degree of deacetylation and acetylation along with other factors such as molecular weight and types of surface modifications [5]. Chitosan has a pKa of 6.5, and it is insoluble in water but soluble in acidic solutions. The protonated species is capable of complexing with a diverse range of anionic biomolecules such as DNA, lipids, and proteins in the form of micro and nanoparticles by means of polyelectrolyte interactions leading to self-assembly. Chitosan NPs are used in a range of drug delivery applications from oral drug delivery to systemic cancer therapy for a variety of payloads including insulin, anticancer drugs, and gene delivery [6]. The biological interactions of chitosan NPs and its derivatives are principally governed by its physicochemical properties, such as size and charge [7,8,9]. The cationic nature of chitosan imparts mucoadhesive properties for mucosal drug delivery applications such as ocular and intranasal delivery. Chitosan NPs have improved the cellular uptake of therapeutics such as anticancer drugs and large molecules such as DNA and proteins [10,11,12,13]. Vaccines formulated with chitosan have high mucosal uptake and the activation of macrophages due to the mucoadhesive and adjuvant activity of chitosan [14]. The fate of chitosan at the cellular level determines its effectiveness in delivering these therapeutic molecules. Consequently, the passage of chitosan through the body and its elimination determines the effectiveness and subsequent toxicity of the therapeutic molecules that it is intended to transport. 

Hence, it is essential to understand the nature of chitosan interactions at both the cellular and tissue levels in order to design effective delivery systems. There have been many reviews of chitosan NPs preparation and application. However, recent reviews on its biological implications and impact on cellular disposition, which will help in the design of advanced formulations, have been informative. Thus, this review paper presents an insight into the various properties that regulate the interactions of chitosan with the cell membrane, its subsequent uptake and passage through the cell, and finally its exocytosis. In addition, the various aspects of in vivo tissue distribution and bioavailability along with its toxicity are discussed.

## 2. Chitosan Cell Interactions

The cell membrane is a multifaceted structure composed of lipids and proteins that provides an effective barrier against the majority of substances. It is important that drug molecules are able to pass this barrier in order to achieve therapeutic activity [15]. The plasma membrane of mammalian cells has a net negative charge attributed to the presence of phospholipids having negative head groups [16]. Hence, a cationic polysaccharide, such as chitosan, can easily attach to the surface of cell membranes by electrostatic interactions, which results in enhanced cellular uptake. Early cellular uptake studies of chitosan solutions with varying molecular weight and degree of acetylation were conducted by Schipper et al. They observed the uptake enhancement potential of chitosan in Caco-2 cells and found that a low degree of acetylation up to 35% and/or a high molecular weight resulted in greater epithelial permeability to mannitol [17]. Similarly, Kotzé et al. were one of the first to report the reduction in transepithelial electrical (TEER) activity with the help of chitosan to enable the transport of peptides across Caco-2 cell monolayers. They compared two salts of chitosan polymer—namely, chitosan hydrochloride and chitosan glutamate—and a chitosan derivative, trimethyl chitosan, and they found chitosan hydrochloride to have the greatest effect in reducing the TEER [18]. Recent mechanistic research on how chitosan affects cellular uptake is discussed in depth in the sections below.

### 2.1. Effect of pH and Zeta Potential on Cell Membrane

The effect of the physicochemical properties of chitosan on the cell membrane has previously been explored by Yue et al. They investigated the effect of surface charge of chitosan NPs on their uptake in eight different cell lines including epithelial cells, endothelial cells, fibroblasts, and blood cells. They observed that positively charged chitosan NPs having a zeta potential of +39.25 ± 2.68 mV had faster cellular internalization kinetics and a greater extent of cellular uptake in all cell lines as compared to neutral (0.51 ± 1.31 mV) and negatively charged (−45.84 ± 2.18 mV) NPs, which were attributed to electrostatic interactions [19]. Introducing surface modification but preserving the positive charge of chitosan NPs retained the cellular uptake-facilitating properties of chitosan. The surface functionalization of gold NPs with chitosan resulted in cationic particles having a zeta potential of +65 ± 1.0 mV. These particles generated rapid internalization within 30 min of treatment of human monocytic THP-1 cells, whereas the negatively charged particles took up to 6 h for apparent cellular uptake [20]. Hu et al. compared the cellular uptake of positively charged chondroitin sulfate chitosan NPs loaded with FITC-BSA having a zeta potential of +16 mV to negatively charged particles with −30 mV. The authors reported that the positively charged particles showed rapid uptake in cells, as demonstrated by a greater fluorescence intensity after 2 h as compared to the negatively charged particles [21]. The surface charge of chitosan NPs is closely associated with the environmental pH. Chitosan has a pKa of 6.5, which allows it to solubilize in acidic pH due to the protonation of amino groups leading to a higher surface charge of the NPs [22,23]. Many NPs have been designed to take advantage of this property of chitosan to selectively release the drug into the desired cell environment, which is especially advantageous for drug delivery to tumors [10,24,25]. For example, pH-responsive NPs using self-assembling polymers made of dimethyl maleic acid–chitosan–urocanic acid with doxorubicin displayed a charge reversal property when transported from a physiological pH of 7.2 to a tumor environment pH of 6.8. The negative zeta potential at physiological pH allowed for better stability during blood circulation with the least non-specific protein adsorption. On the other hand, a switch to a positive zeta potential in the tumor microenvironment allowed enhanced cellular uptake and rapid release of doxorubicin from the NPs [12]. In another research study, chitosan was used as a pH-sensitive stealth coating for the delivery of paclitaxel to tumor cells. NP formulations with PLGA and low molecular weight chitosan (50–190 kDa) showed better association with SKOV-3 and NCI/ADR-RES cells at a pH of 6.2 due to the positive charge of the particles as compared to those at a pH of 7.2 [24]. This pH-sensitive property of chitosan allows it not only to better associate with cells and provide enhanced cellular uptake but also to target specific microenvironments in and around cells. 

### 2.2. Effect of Chitosan on Cell Adhesion

The passive attachment of cells to a static substrate, such as culture flasks and Petri dishes, is attributed to specific membrane integrin binding interactions providing mechanical linkage between the intracellular actin and the extracellular matrix [25]. Chitosan membranes have been used to recover cultured cells from substrate without any enzymatic treatments [26]. They demonstrated that up to 90% of cells detached from a chitosan-coated surface when the medium pH was changed from the pH in which the cells were incubated for 24 h (7.2) to a pH of 7.65 for one hour with up to 95% cell viability. This pH-induced detachment was observed in five different types of cell lines: namely, HeLa, primary corneal fibroblasts, HaCaT, H1299, and NIH-3T3 cell lines. This observation can be explained by the deprotonation of chitosan at higher pH, which also leads to the desorption of fibronectin from the substrate, thus further increasing the detachment of cells [26]. The charge-dependent interaction of chitosan with the cell membrane to control cell adhesion behavior has been exploited recently. Stronger adhesion was demonstrated for the PC-3 prostate cancer cell line to hyaluronic acid–chitosan films from pH 3.0–5.0 [27]. Better adherence of bovine chondrocytes to chitosan films neutralized with sodium hydroxide was observed, which impacted cell proliferation and adhesion on scaffolds [28]. The enhanced absorption of fibronectin on collagen-chitosan film surfaces aided in the better adhesion of PC12 cells [29]. The degree of acetylation of chitosan has been reported to influence its impact on cell adhesion and proliferation properties due to changes in hydrophobicity and the number of protonated groups available for cell adhesion. A greater degree of acetylation of chitosan used in films displayed increased hydrophobicity and a lower surface charge, leading to decreased adhesion of fibroblast and chondrocytic cells [30]. Similarly, olfactory epithelial cells displayed better cell compatibility and proliferation activity at a higher degree of deacetylation of up to 85% in turn, indicating that a lesser acetylation of chitosan was beneficial for cell growth and adhesion [31]. The effect of chitosan on cell adhesion has found various applications in tissue engineering such as scaffolds for bone and cartilage regeneration [32], peripheral nerve regeneration [33], skin grafting [34,35], wound healing [36,37], and 3D cell cultures [38,39].

### 2.3. Effect on Tight Junctions

Chitosan has been used as a permeation enhancer due to its unique property to transiently open the epithelial tight junction to allow the penetration of hydrophilic molecules such as insulin [40,41]. Epithelial cells form an effective protective barrier with intercellular contacts through tight junctions, adherin junctions, and desmosomes. Tight junctions are composed of a combination of various proteins such as occludens, claudins, and junctional adhesion molecules found between epithelial and endothelial cells, which protect organs from the outside environment and help to maintain homeostasis [42]. Tight junction integrity is typically indicated by changes in the transepithelial electrical resistance. Tight junction proteins have an intracellular and extracellular portion that are responsible for maintaining intercellular communication and the transport of small molecules, ions, and water via active transport [43,44]. The intestinal tight junctions play a pivotal role in protecting the body against stress stimuli such as infections and inflammation [42]. A mixture of carboxymethyl chitosan with chitosan NPs, creating a hydrogel, was found to be effective for the delivery of insulin across the intestinal barriers. The NPs not only had gastric protective ability but were able to selectively release insulin in the intestinal medium in vivo. Similarly, the chitosan-related redistribution of claudin-4 from the cell membrane to the cytosol leading to a reversible opening of tight junctions in Caco-2 cells was also observed [40,45]. Thus, it is clear that the penetration of chitosan via tight junctions has multiple mechanisms. 

### 2.4. Chitosan and Transepithelial Electrical Resistance (TEER)

The effect of chitosan on paracellular permeability has been well established. Transepithelial electrical resistance measurements are a valuable tool that provide an insight into the cell membrane permeability and integrity. TEER is the measurement of electrical resistance across the cell monolayer, which reflects the integrity of the tight junctions and cellular barriers [46]. Chitosan solutions applied to cells induced a dose-dependent decrease in TEER of Caco-2 cell monolayers of up to 83% at 0.5% (*w/v*) concentration, which was independent of the pH of the medium [47]. In turn, this reduction in electrical resistance is indicative of the simultaneous increase in membrane permeability. Chitosan, either in solution or prepared as NPs, has shown a decrease in TEER and an increase in cell permeability via the tight junction pathways by the displacement of the zonula occludens-1 proteins in Calu-3 cells [48]. TEER measurements can be conducted across a wide range of spectrum frequencies and in real time without causing any damage to live cells. However, variations can arise due to changes in temperature, the passage number of cells, the type of medium, and the degree of cell confluency [46]. The various physical and chemical properties of chitosan affecting its interactions with cells have been summarized in Table 1.

## 3. Pathways of Cellular Uptake

**Figure 1:**
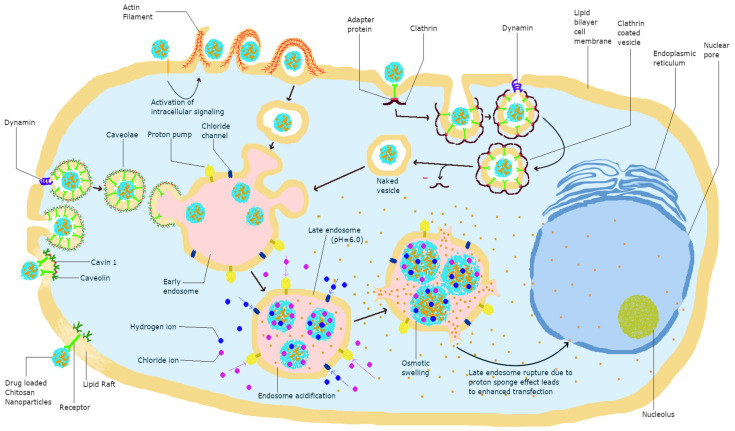
Modes of cellular uptake and eventual fate of chitosan in cells.

Charged biomolecules are mainly transported across the cell membrane through an active endocytosis transport mechanism [49]. Chitosan is known to be transported mainly via this mechanism and specifically, the endocytosis of chitosan NPs involves the two major pathways of phagocytosis and pinocytosis. Furthermore, chitosan uptake by pinocytosis can be divided into caveolin-mediated, cadherin-mediated, and clathrin-mediated uptake. Phagocytosis is the uptake of particles larger than 250 nm, whereas pinocytosis involves the pathways of cellular uptake of chitosan and is dependent on its size, charge, and surface modification. Clathrin-mediated cellular uptake involves transport via clathrin-coated pits, leading to internalization followed by integration into late endosomes delivering their cargo to lysosomes [50]. Caveolae-mediated endocytosis utilizes cholesterol-rich, flask-shaped invaginations in the plasma membrane, which internalize from the plasma membrane with the help of GTPase dynamin, delivering cargo to lysosomes [50,51]. The different interactions of chitosan with cells are depicted in Figure 1, and further visual representation of chitosan NPs with cells using confocal microscopy can be found in the following references [9,52,53,54].

### 3.1. Effect of Size and Charge on Uptake Pathway

**Figure 2:**
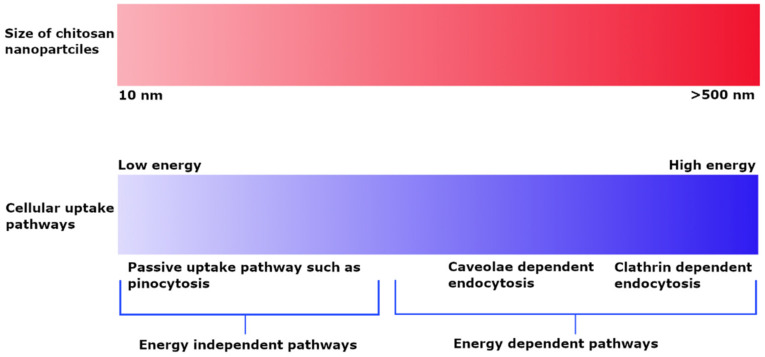
Effect of particle size on cellular uptake pathways depicting energy dependent uptake of larger sized NPs and passive uptake of small NPs.

The cellular uptake of chitosan NPs is based on their size and is dependent on the type of cell line and experimental conditions. PLGA NPs coated with chitosan having a particle size of up to 200 nm demonstrated more extensive cellular uptake into A549 cells as compared to 1000 nm particles and suggested an energy-dependent clathrin-mediated endocytic process [55]. The cellular uptake of chitosan particles loaded with ovalbumin and FITC–bovine serum albumin on bone marrow-derived dendritic cells and the RAW 264.7 mouse macrophage cell line indicated that uptake was dependent on size rather than incubation time and concentration. Particles in the 1 µm range were taken up to a greater extent by both macrophage and dendritic cells as compared to smaller (300 nm) and bigger (3 µm) particles [56]. In contrast, FITC-labeled chitosan NPs having a particle size of about 250 nm exhibited clathrin-mediated endocytosis; phagocytosis occurred to a smaller extent in macrophages [9]. The uptake of smaller chitosan NPs in the 25 nm range in L929 fibroblast cells indicated a passive uptake, whereas the uptake of larger NPs of up to 150 nm was more energy-dependent in the presence of sodium azide. Both NP sizes showed caveoli- and lipid raft-dependent endocytic processes [57]. Observation of the dependence of the uptake pathway on charge indicates that non-phagocytic cells preferentially uptake cationic NPs, although charge density and hydrophobicity also have a role. Both anionic and cationic charged chitosan NPs prefer clathrin-mediated endocytosis, although no general rule applies [58]. He et al. investigated the phagocytic and non-phagocytic uptake of rhodamine-labeled negatively charged carboxymethyl chitosan NPs and positively charged chitosan hydrochloride-grafted NPs with particle sizes of 300 nm and 500 nm in murine peritoneal macrophage cell lines. Positively charged particles displayed greater phagocytic uptake as compared to negatively charged particles. Similarly, the non-phagocytic uptake of these particles favored positively charged NPs as compared to negatively charged ones, although the extent of uptake was highly dependent on the type of cell line. Furthermore, the uptake process was highly energy dependent, as demonstrated by the observation of significant inhibition of uptake at 4 °C. Clathrin and caveolae-mediated cell uptake processes were also common [59]. The effect of particle size on uptake pathways is summarized in Figure 2.

### 3.2. Effect of Hydrophobicity/Hydrophilicity on Uptake Pathway

The incorporation of hydrophobic chains to polymers can enhance cellular uptake for the delivery of genes as well as uptake by tumor cells [60,61]. This enhanced uptake is mainly attributed to the increased hydrophobic interactions with the lipid cell membrane, although cellular uptake still takes places via various mechanisms including clathrin- and caveoli-mediated pathways. Hydrophobic modifications on glycol chitosan were utilized by Nam et al. for targeted delivery to tumors. The uptake in HeLa cells by hydrophobically modified chitosan NPs having an average particle size of 310 nm involved both clathrin- and caveoli-mediated pathways in addition to micropinocytosis, with all pathways displaying an additive effect on the cellular uptake. Therefore, this report suggested that all these pathways were involved [61]. Hydrophobically modified *N-*palmitoyl chitosan with a particle size of about 200 nm indicated non-clathrin-mediated uptake at lower degrees of hydrophobic substitution (e.g., 5%, 10%, and 15%), whereas macropinocytosis was the preferred route of uptake at higher degrees of substitution of 15% and 20%. The caveolae-mediated route of uptake was the most affected as the degree of hydrophobicity of NPs increased, which indicated that lipid raft-mediated caveoli was the most preferred route at high hydrophobicity [62]. 

Hexanoic acid and monomethoxy-PEG modifications on chitosan to form pDNA polyplexes were utilized for the efficient delivery of genes to HEK 293 cells. These polyplexes with a diameter of less than 200 nm and positive zeta potential showed predominantly clathrin-mediated cellular uptake [63]. Similarly, both hydrophilic and hydrophobic modifications of chitosan with linoleic acid and poly (β-malic acid) forming a doubly grafted chitosan formic complex with pDNA (mean diameter <200 nm) indicated a process dominated by clathrin-mediated uptake [64]. 

### 3.3. Covalent Modifications

Chitosan and its derivatives have been exploited to fabricate targeted drug delivery systems to achieve the desired effect of drug at lower concentrations [65] by attaching various ligands, which include peptides, vitamins, hormones, carbohydrates, and proteins, as shown in Table 2. Receptor-mediated endocytosis is the primary route of uptake for targeted systems functionalized with ligands [66,67,68,69]. Overexpression of the receptors on specific cells in certain disease states facilitates the selective binding of chitosan and its derivatives by targeted drug delivery [11,13,70,71], which leads to enhanced drug delivery to specific target sites. Kai Jiang et al. utilized the pH-sensitive nature of chitosan polymers to deliver a hydrophobic drug, namely ursolic acid, into endosomes (pH < 5.5) and reported the suppression of cancer cell growth [72]. The derivatization of chitosan polymers broadens opportunities such as dual-ligand functionalization [73], co-polymerization to develop amphoteric block co-polymers [11], co-delivery of drug molecules and oligonucleotides [68], and improving solubility [70]. Zhu et al. reported that chitosan derivative *N*-succinyl-*N*′-octyl chitosan self-assembles to form micelles due to its amphipathic nature, and conjugation with folic acid imparts a targeting nature to the micelles [74]. Further modifications of chitosan affecting pathways of cellular uptake are summarized in Table 2.

## 4. Intracellular Disposition of Chitosan

The endocytosis mechanism determines the intracellular fate of NPs. Endocytosis transports the NPs within vesicles where they are released into the subcellular compartments such as lysosomes, mitochondria, or Golgi bodies. Nevertheless, it is important that the NPs are able to release their cargo once transported inside the cells. Once released into the cells, the NPs must be able to travel through the cytoplasm to reach their target organelles, which is dependent on the physicochemical properties of the NPs such as size, charge, and physicochemical properties [84].

### 4.1. Endosomal Escape

One essential requirement of gene therapy is the efficient escape of NPs from endosomes to deliver drugs into the cytoplasm, which can be later taken up by the nucleus for effective gene transfection. The enzymes within lysosomes can cause significant degradation of the encapsulated molecules, leading to reduced efficiency. Chitosan NPs have shown the potential for enhanced endosomal escape due to the phenomenon well-known as the “proton sponge effect.” The acidic environment within endosomes causes amine groups on chitosan, which have a pka of 6.5, to become increasingly protonated in the endosomes, thus leading to a high influx of water and chloride ions to balance the charges. This influx causes the lysosomes to swell and rupture, releasing their contents into the cytoplasm [85]. However, chitosan on its own has a poor buffering capacity [86], as demonstrated by the uptake of poly (methacrylic acid) surface decorated on 10-hydroxy camptothecin-loaded CTS NPs having a positive charge at pH 6.5. The positive charge of chitosan NPs facilitated protonation at endosomal pH 6.5, leading to endosomal rupture due to the proton sponge effect. This was confirmed by the assessment of the integrity of the endosomal membranes, as indicated by a characteristic shift in the fluorescence of the endosomal marker acridine orange after the rupture of the endosomes [87]. Similarly, various modifications on chitosan for gene delivery have been utilized for efficient endosomal escape: grafting imidazole moieties on the chitosan backbone from DNA–polymer complexes for improved transfection in HEK 293 cells and HepG2 cell lines [88]; the addition of histidine to the chitosan backbone, forming complexes with pDNA in HEK cell lines and enhancing the buffering capacity of chitosan to enhance endosomal escape [89]; and enhancing the buffering capacity of chitosan with the help of alkyl amino acids for delivery of the p53 gene in HEK and A549 lung cells [90]. 

### 4.2. Co-Localization with Lysosomes

Molecules endocytosed into cells reach lysosomes for degradation via early and late endosomes. After NPs are taken up by different mechanisms, they reach the lysosomes where their preferential uptake is determined by their size and surface properties [8]. Lysosomes contain various degradative enzymes and are the most acidic organelles, with a pH ranging from 4.5 to 5.5, which causes enzymatic degradation of NPs. NPs are designed to target lysosomes in order to treat lysosome-related diseases or to escape lysosomal degradation to deliver materials into areas of the cell [91,92]. Chitosan causes the rupture of lysosomes by a process similar to that for endosomes through the proton sponge effect, as shown by Fong et al., where they studied the effect of acetylation of chitosan to produce lysosomal rupture and subsequent inflammatory response. They found that the lysosomal disruption was dose dependent with lower doses producing a mild disruption, whereas higher doses of chitosan produced higher levels of lysosome disruption in U937 cell lines differentiated to macrophages [93]. In another study, the cationic charge of chitosan enabled efficient lysosomal escape in HEK cells to increase the chitosan–pDNA polyplex transfection efficiency. Free chitosan, when pre-incubated in cells before allowing transfection, led to chitosan localizing in the lysosomes, which increased the transfection of DNA polyplexes previously having a low transfection efficiency [94]. In contrast, hyaluronic acid-conjugated chitosan NPs have been shown to entirely escape lysosomal uptake in corneal and conjunctival cell lines [95].

### 4.3. Nuclear and Perinuclear Localization

Drug delivery to the nucleus involves overcoming the nuclear envelope barrier and transport through the nuclear pore complexes, which are perforations in the nuclear envelope. Transport through the nucleus is mainly dependent on size, where smaller molecules are transported across by passive diffusion and larger molecules are sorted via oligopeptide receptor-mediated active transport using nuclear localization signals (NLS) [96]. Nuclear localization is essential for drugs exhibiting their main mechanism of action at this site [97]. Tammam et al. have indicated that chitosan NPs having a smaller size of up to 25 nm displayed up to a five-fold increase in nuclear localization rates of albumin–FITC-loaded NPs as determined by FRET microscopy, whereas larger NPs of up to 150 nm required modification with an octapeptide nuclear localization sequence to produce nuclear localization of up to 3.7-fold improvement in murine fibroblast L929 cells [57]. Furthermore, an NLS peptide sequence associated with a chitosan–DNA complex improved the transfection efficiency up to 74-fold as compared to a plain chitosan–DNA complex in HeLa cells. These ternary complexes had a particle size of up to 500 nm and zeta potential of about +12 mV [98]. The perinuclear localization of NPs provides proof of endosomal/lysosomal escape and potential uptake by the nucleus. Additionally, chitosan NPs persist in cells for a long time and can become entrapped inside the nucleus subsequent to nuclear envelope reassembly at the end of mitosis; this temporal advantage also helps to target drugs into the nucleus, irrespective of size of the NPs and nuclear localization signal [52]. Additional examples include various other covalent modifications on chitosan: alkyl glyceryl chitosan NPs in mouse brain capillary endothelial cells [99]; tamoxifen-loaded lecithin/chitosan NPs in Caco-2 cells [100]; and glycol chitosan-β cholanic acid NPs loaded with protoporphyrin IX for targeted photosensitizer delivery in squamous cell carcinomas [101]. These approaches have been investigated to localize chitosan NPs into the perinuclear region, therefore enhancing their uptake into the nucleus.

### 4.4. Mitochondrial Metabolism

Chitosan NPs have been employed for the mitochondrial targeting of drugs; however, their mechanism of targeting this intracellular site is unclear. Targeted delivery to the mitochondria has been employed in the treatment of several tumors. NPs can take advantage of the difference in mitochondrial membrane potential and release of reactive oxygen species (ROS), causing rupture and the release of mitochondrial DNA and ultimately causing cell death. The mitochondrial membrane potential and ROS generation have an exponential correlation; hence, even a small difference in membrane potential can lead to a considerable increase in ROS [102]. Chitosan NPs having a size of about 65 nm and zeta potential of +52 mV caused a dose-dependent decrease in mitochondrial membrane potential in human gastric carcinoma MGC803 cells causing disruption of the mitochondrial membrane and subsequent necrosis [103]. Similarly, chitosan NPs having an average particle size of 84 nm and charge of +17 mV caused a decrease in mitochondrial membrane potential and generation of ROS to induce cell death in hepatocellular carcinoma SMMC-7721 cells [104]. Covalent modifications of chitosan to aid in its mitochondrial targeting have also been investigated, such as *N-*glycyrrhetinic acid-PEG-chitosan and *N-*quaternary ammonium chitosan NQC loaded with brucine, which indicated mitochondrial targeting in HepG2 cells [105]. Triphenyl phosphine-conjugated chitosan NPs, hyaluronic acid-coated chitosan NPs, and glycol chitosan polymerized with dequalinium have all been used; however, in these cases, chitosan was utilized as an aid for efficient cellular uptake, whereas the mitochondrial targeting moieties were triphenyl phosphine, hyaluronic acid, and dequalinium, respectively [106,107,108]. Conversely, carboxymethylated chitosan and chitosan-coated iron oxide NPs have been used to prevent mitochondrial stress and hydrogen peroxide-induced cell death in Schwan cells in addition to mitochondrial membrane protection with reduced ROS generation in HeLa, A549, and HEK 293 cell lines, respectively [109,110].

### 4.5. Exocytosis of Chitosan Nanoparticles

Although chitosan is considered relatively safe, its exocytosis from cells determines its therapeutic toxicity and biosafety [111,112]. The exocytosis of NPs from cells is affected by the various physicochemical properties of NPs such as size, shape, surface modification, concentration, and incubation time with cells [111,112]. As demonstrated by Park et al., the exocytosis of *N-*acetyl histidine chitosan NPs was dependent on the pre-incubation time with nanoparticles before the removal of free nanoparticles from the media. With short incubation times, the self-assembled *N-*acetyl histidine chitosan NPs dissociated in the acidic endosomes. However, with longer incubation times of 6 h, the exocytosis of NPs was observed from HeLa and A549 cells [113]. Chitosan-coated PLGA NPs again showed an incubation time-dependent exocytosis of NPs from mast cells with plain PLGA NPs displaying higher exocytosis as compared to chitosan PLGA NPs [114]. This strategy may thereby promote entry of the payload in the cytosol. On the other hand, chitosan-coated ultra-small superparamagnetic iron oxide NPs have been designed for long-term retention in a variety of cell lines with NPs observed in cells for up to 10 days [115]. Doxorubicin-loaded deoxycholic acid-modified carboxymethyl chitosan NPs indicated time-dependent accumulation in MCF-7 cells with faster uptake within 6 h of incubation and longer retention time in cells after 24 h as compared to free doxorubicin [116]. Thus, it can be concluded that chitosan helps prolong the retention time of these types of particles within cells.

### 4.6. Cytotoxicity upon Cell Internalization

**Figure 3:**
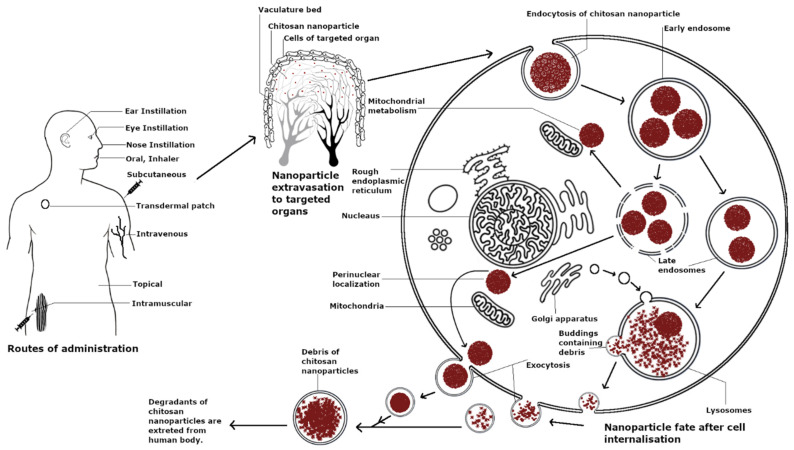
Lifespan of chitosan nanoparticles.

Chitosan is considered a biocompatible material. Chitosan and chitosan-coated NPs have displayed no toxicity in a variety of cell lines such as MDBK cells, Colo 205 cells, human antigen-presenting cells, respiratory epithelial cells Calu-3 and A549, and 3T3 fibroblast cells [117,118,119,120]. Similarly, amino acid modification on glutaraldehyde cross-linked chitosan NPs, such as lysine and glutamic acid modifications, induced no cytotoxicity even at concentrations up to 250 µg mL^−1^ and suppressed the toxicity of copper oxide-loaded NPs in HepG2, A549, and RAW264.7 cells [121]. However, chitosan cytotoxicity has been shown to be dependent on the cell line type, particle size, and concentration and particularly favors cancerous cells as compared to non-cancerous cells. Fast-growing cancer cells, namely COS-1, showed higher sensitivity to chitosan-coated PLGA NPs as compared to epithelial A549 and Calu-3 cells in terms of concentration and incubation times; however, the charge of particles did not affect the toxicity in COS-1 cells [122]. Similarly, chitosan NPs displayed an IC_50_ of 10 µg/mL after 24 h in human T lymphocyte acute leukemia cells and were significantly cytotoxic at 50 µg/mL but displayed no cytotoxicity at the same concentration in human embryonic kidney HEK 293 cells. The higher toxicity of chitosan NPs in tumor cells as compared to non-cancerous cells can be attributed to various factors such as differences in the mitochondrial membrane potential, increase in mitochondrial dehydrogenase activity, the generation of ROS, and enhanced uptake in tumor cells [123]. Chitosan NPs (50–100 nm) were recently used for immune cell anti-tumor therapy in γδ T cells, which are innate-like T lymphocytes and are an early source of IFN-γ. The NPs functionally upregulated Vγ9Vδ2 T cells, induced their activation, and enhanced tumor cytotoxicity through α-tubulin cytoskeleton rearrangement and polarization [124]. The cellular lifespan of chitosan is summarized in Figure 3. 

## 5. In Vivo Tissue Distribution and Bioavailability of Chitosan Nanoparticles

The effect of chitosan at the biological interface involves not only its effect on cells but also behavior upon intake through various routes. Early pioneering research on chitosan particles biodistribution was reported by Richardson, Suzuki, Onishi, and Banerjee et al. [125,126,127,128] and extensively reviewed by Thanou et al. [129]. They found that following administration into the body, chitosan particles rapidly distribute and are quickly cleared, especially after intravenous administration. However, the size and charge of chitosan NPs, its MW, degree of acetylation, covalent modifications, and the extent of protein corona formation all play a major role in its ability to achieve long circulation times in blood to prevent premature elimination of drugs from the body. The significance of chitosan NPs in preventing the premature elimination of encapsulated drugs from the body has been summarized in Table 3. In turn, these properties affect the safety of these NPs for clinical applications [129].

### 5.1. Effect of Protein Corona

The fate of NPs upon entering systemic circulation is governed by the protein corona surrounding it. Protein coronas are characterized by a dynamic “soft corona” and a long lasting “hard corona”, which remains strongly adsorbed onto the nanoparticle surface. The composition of the protein corona affects how NPs interact with platelets and blood cells in addition to how they influence various aspects of nanoparticle design such as drug release, active targeting, and surface functionalization [7,138,139]. Negatively charged serum proteins are preferentially adsorbed onto the surface of positively charged NPs due to electrostatic interactions [140]. Comparably, positively charged chitosan NPs have been demonstrated to attract large quantities of protein on their surface, as evidenced by Almalik et al. In addition, they demonstrated that decreasing the zeta potential from cationic to anionic, by coating the chitosan nanoparticle surface with negatively charged hyaluronic acid, significantly decreased the non-specific protein interactions of the NPs [141,142]. Furthermore, chitosan-coated PLGA NPs displayed higher affinity to serum immunoglobulins due to the innate immunogenic response of positively charged chitosan as compared to heparin-coated PLGA NPs, which displayed higher affinity to serum albumin [143]. Abouelmagd et al. maintained that despite the high serum protein adsorption on the surface, chitosan NPs maintained the pH sensitivity necessary for efficient drug delivery. They prepared PLGA NPs coated with low MW chitosan for the delivery of paclitaxel in SKOV-3 human ovarian cancer cells. PLGA NPs were produced either from PLGA pre-conjugated to LMW chitosan, or the PLGA NPs were formed first, which was followed by surface conjugation with polymerized dopamine and subsequent incubation with LMW chitosan, effectively spacing the chitosan away from the surface. This reduced the hydrophilicity of the NP to better enable hydrophobic drug loading. The LMW chitosan-modified NPs had a protein corona increasingly enriched with immunoglobulins in addition to serum albumins. They were more effective for enhancing cell uptake under acidic compared to neutral pH conditions, thus demonstrating pH sensitivity. When formulated as microparticles (1–3 μm), this approach demonstrated that the surface modification with LMW chitosan reduced phagocytic uptake by J774A.1 mouse macrophages, which was possibly due to the hydrophilicity imparted by the chitosan coating [144]. In another strategy to reduce RES uptake, a recent study of the protein corona formation and biodistribution of chitosan and its polyelectrolyte complex with carboxymethyl dextran and thiolated dextran indicated that dextran modification of chitosan resulted in a reduced protein corona and low liver uptake. A net negative surface charge was associated with lower protein binding, and the protein corona was dominated by protein C, hemoglobin subunits, and apolipoproteins, which play a role in the induction of uptake of NPs by macrophages as well as their eventual phagocytosis and biodistribution [145].

### 5.2. Effect of Mucosal Routes of Administration

The cationic surface properties of chitosan NPs allow it to bind strongly to negatively charged mucosal surfaces, making it an excellent candidate for mucosal routes of administration such as oral, intranasal, ophthalmic, and vaginal routes. Chen et al. prepared chitosan NPs for the oral delivery of heparin and followed the biodistribution of ^99m^Tc-labeled chitosan NPs upon oral administration. They found that the mucoadhesive property of chitosan allowed these NPs to predominantly accumulate in the intestinal mucosa with a gastric retention time of 8 h and the majority of radioactivity observed in the colon after 24 h. There was minimal systemic absorption and accumulation in internal organs [146]. Similarly, colon-targeted curcumin-loaded chitosan NPs coated with Eudragit FS 30D were found to retain highly in the colon with little accumulation in other organs [147]. Navarro et al. reported less than 1% accumulation of chitosan NPs in the spleen, liver, kidneys, heart, lungs, and brain after oral administration of fluorescent tagged FITC–PLGA–chitosan NPs daily for 7 days and attributed these findings to chitosan’s mucoadhesive properties and the rapid elimination from the systemic circulation [148]. Similarly, the mucoadhesiveness of chitosan leads to longer retention time in the nasal cavity. Gartziandia et al. reported NPs accumulation in the brain after 30 min of intranasal administration, which was observed by fluorescence imaging of DIR-labeled chitosan–lipid nanocarrriers, which were retained after 24 h. Additionally, fluorescent NPs were found to concentrate mainly in the lungs and to a smaller extent (less than 1%) in liver, spleen, and kidneys due to non-specific mononuclear phagocyte capture. Nanoparticles were present in the nasal cavity for up to 24 h, allowing additional time for them to reach the brain [149]. Similarly, chitosan–lecithin NPs for brain targeting loaded with phenytoin demonstrated relatively high accumulation in brain and low phenytoin levels in plasma after intranasal delivery as compared to intraperitoneal injection, which produced initial high plasma levels before crossing the blood–brain barrier [150]. The intraocular delivery of chitosan inserts radiolabeled with Tc-99m resulted in the retention of NPs for up to 6–8 h at the site of instillation in the eye, after which the formulation accumulated in the gastrointestinal tract, mainly the large intestine, via nasolacrimal clearance [151,152].

### 5.3. Effect of Surface Modifications

The surface modification of chitosan NPs allows them to target the desired site of action and has been utilized for various applications depending on the type of modification such as tumor targeting, oral delivery for GIT uptake, ocular delivery, lymphatic system targeting, and prolonged systemic circulation. Kamiyama et al. first reported the biodistribution of modified chitosan to form water-soluble chitosan derivatives *N-*succinyl chitosan and glycol chitosan for tumor sarcomas targeting. The fluorescent isothiocyanate FITC-modified glycol chitosan solutions displayed higher partition into the tumors as compared to other organs, whereas FITC-labeled succinyl chitosan solutions displayed longer blood residence time and a greater accumulation rate into the tumor [153]. Li et al. prepared dual ligand-decorated galactosylated chitosan with glycyrrhetinic acid for hepatocellular carcinoma targeting. The NPs specifically accumulated at the tumor site followed by liver, spleen, and kidneys [154]. The modification of chitosan NPs with the thermos-sensitive polymer poly (*N-*vinylcaprolactam) and a cell-penetrating peptide (RLYMRYYSPTTRRYG) for targeting triple negative breast cancer with doxorubicin collected extensively in the tumor region and also to a lesser extent in the liver, kidneys, and spleen. The modified chitosan polymer had a phase transition temperature that was clinically achievable for tumor heating (<40 °C), enhancing local drug release. Furthermore, the slightly acidic tumor environment promoted dissolution of the chitosan NPs and thus triggered release from these dual pH/thermo-responsive NPs [155]. Carboxymethyl-β-glucan/chitosan NPs have been recently used to target vaccines to the lymphatic system. These NPs having a size of about 150 nm and charge +30 mV were loaded with ovalbumin and showed significant lymphatic accumulation due to the size and charge of the NPs and the ability of carboxymethyl-β-glucan to interact with antigen-presenting cells [156]. Surface-modified negatively charged poly(methacrylic acid) conjugated chitosan NPs loaded with 10-hydroxy camptothecin (HCPT) significantly increased blood circulation time from 12 h to 24 h and reduced blood clearance (CI) from 30 to 6 mL/h as compared to the non-surface modified NPs. Additionally, the t_1/2_ of the surface-modified NPs was longer by 4.37-fold and that of the non-surface modified NPs was longer by 2.48-fold compared to free drug [87]. Other examples of chitosan modifications for various targeting applications are summarized in Table 4. These examples provide evidence of the versatile nature of chitosan modified to suit multiple targeting applications.

### 5.4. Effect of Physical Properties on Biodistribution

So far, we have discussed the effect of particle size and charge and hydrophobicity on chitosan NP cellular uptake and toxicity and given examples of modifying the surface properties. These attributes also impact systemic biodistribution. Onishi et al. studied the biodegradation and distribution of solutions of 50% deacetylated chitosan labeled with FITC upon intraperitoneal administration. The FITC–chitosan solution was found to accumulate in the kidneys and rapidly eliminate in urine after 14 h of administration with little accumulation in liver and spleen [127]. Furthermore, Banerjee et al. reported the biodistribution of 100 nm chitosan NPs which were 100% cross-linked with glutaraldehyde labeled with ^99m^Tc. They observed a rapid and maximum accumulation of the NPs in the liver within 30 min of intravenous administration followed by other organs, which subsequently declined over 4 h. They also observed increased radioactivity in the stomach after 4 h, indicating dissociation of the radioactive complex. They attribute this effect to the small particle size of chitosan NPs and hydrophilicity of the polymer [128]. Zhang et al. analyzed the biodistribution of *N-*octyl-O-sulfate chitosan micelles with 65 kDa chitosan loaded with paclitaxel having a size of about 200 nm and negative surface charge of −28.8 mV, following intravenous administration. They observed rapid and wide distribution to all organs of the body with maximum accumulation in spleen followed by liver after 8 h of administration, with the longest retention time in the lungs [166]. Similarly, Bachir et al. compared chitosan (MW 11 kDa) NPs conjugated with PEG at various degrees of substitution loaded with methotrexate having a particle size of about 110–171 nm and zeta potential +7–35 mV found that non-PEGylated chitosan NPs accumulated significantly in the liver and spleen at 24 h following intravenous administration and to a lower extent in lungs, kidneys, and heart. In contrast, PEGylated chitosan NPs distributed primarily to the kidneys [167]. Intraperitoneal injection of FITC-labeled carboxymethyl chitosan revealed similar high uptake in the liver of 300 kDa molecular weight chitosan, whereas unspecified chitosan degradation products of about 45 kDa were found in the urine [168]. Feng et al. prepared pH-responsive NPs of chitosan and O-carboxymethyl chitosan of MW 10–12 kDa having a size of about 270 nm and negative zeta potential (−33 mV) loaded with doxorubicin for oral administration. These NPs were capable of selectively releasing doxorubicin into the intestinal environment and enhanced transport across the epithelial tight junctions. They achieved prolonged accumulation and retention in the liver, spleen, and lungs. This controlled release property of the NPs reduced the cardiac and renal toxicity of doxorubicin. Thus, chitosan NPs have the liver and spleen as the significant sites of accumulation, similarly to other types of NPs, suggesting that their reticuloendothelial uptake dominates biodistribution [162].

## 6. Systemic Toxicity and Elimination of Chitosan Nanoparticles

Designing a drug delivery system requires in vivo toxicity studies to assess their applicability, especially for nanoparticulate systems. Chitosan is widely considered a nontoxic and biologically compatible polymer; however, chitosan NPs need to be scrutinized for their safety potential. A review of chitosan’s in vivo toxicity by Thanou et al. previously found chitosan to be relatively nontoxic at doses needed for therapeutic delivery. Some toxicity was observed at high doses, but they generally concluded chitosan to be safe for in vivo applications [129]. Sonin et al. recently assessed various factors such as the hemolytic activity and acute and sub-acute toxicity of chitosan NPs over a 14-day period. Chitosan MW 138 kDa NPs of about 100 nm in size having a weak positive charge of +10 mV were administered as a single intravenous dose of 1, 2, and 4 mg/kg of chitosan in mice and observed over 2 weeks. The NPs displayed negligible in vivo antiplatelet activity and ex vivo anticoagulant activity with the least toxicity in cardiomyocyte cell cultures. Upon IV administration, about 93% of the NPs accumulated in the liver with about 6% accumulation in lungs within 30 min of administration with no organ cytolysis nor necrosis observed after 14 days. They found small granulomas in the lungs consistent with the physiological macrophage containment of foreign bodies and by which the authors suggest a slow degradation process. Overall, these results suggest that chitosan NPs and chitosan degradation products are well tolerated and naturally eliminated at least for those NP with near-neutral surface charge [169]. Nadesh et al. established that chitosan NPs having a high surface charge of more than +30 mV produced hematotoxicity, whereas NPs having a highly negative charge of about −40 mV resulted in their phagocytosis and rapid elimination. Hence, the biocompatible range of charge for chitosan NPs is +15 to −30 mV [59,170]. This also highlights the need to conduct hemolysis assays for novel chitosan NPs intended for IV administration. Sonaje et al. studied the toxicity of pH-sensitive chitosan NPs conjugated with poly-γ-glutamic acid in the presence of MgSO_4_ and TPP for the oral delivery of insulin (size ≈ 200 nm, charge +25 mV). Blank chitosan NPs (100 mg/kg) upon daily administration over 14 days displayed no signs of toxicity such as diarrhea or fever and no mortality at the end of the study, indicating no oral toxicity [171]. Shan et al. studied the acute and chronic toxicity of angiopoietin-2 with small interfering RNA plasmid chitosan magnetic NPs after intravenous administration of different doses and observed them over 14 days. Upon observation of acute toxicity, the mice in the middle and high dosage groups (254.6, 424.2, and 707.0 mg.kg^−1^.d^−1^) exhibited short-term reduced activities and heavy breathing attributed to partial lung congestion due to lung phagocytosis as compared to the low-dosage groups (91.6, 152.8 mg.kg^−1^.d^−1^) and control group. Chronic toxicity studies revealed no significant impact on the well-being of the animals and no deaths. However, chronic lung congestion was found upon morphological observation of lungs after 14 days of daily NP administration with a high number of white blood cells in the middle and high-dose groups (70.70 and 353.50 mg.kg^−1^.d^−1^) as compared to the control and low-dose group (35.35 mg.kg^−1^.d^−1^) indicative of the lung being the target organ for the elimination of these magnetic NPs upon IV injection [172]. However, no other studies reporting lung congestion were found within the scope of the literature search for this review. Hence, most studies have demonstrated chitosan as minimally toxic except for pulmonary congestion on IV administration of very high doses, justifying its selection as a safe material in drug delivery. The high pulmonary accumulation of chitosan is likely due to its degradation primarily by the enzyme lysozyme, which is abundant in lungs and also highly expressed in hematopoietic cells, granulocytes, monocytes, and macrophages. However, for novel chitosan nanoparticles, it would be prudent to conduct lung histopathology studies in the course of early toxicological evaluations as a part of dose–response studies. Although there are no conclusive in vivo studies on the complete degradation and elimination profiles of chitosan, most studies conclude that chitosan is biodegradable. In vitro degradation studies of chitosan suggest that the rate and degree of degradation depends on various factors such as the concentration of enzymes, molecular weight of chitosan, degree of acetylation, type and concentration of cross-linking agents, and pH of the medium [30,173].

## 7. Current Clinical Investigations and Challenges

Chitosan has been used in various marketed products in the food and beverage industry. The chief markets for nutraceutical formulations of chitosan are Asia including Japan, Korea, and China with the USA and Europe quickly expanding as well. Chitosan products include ChitoClear^®^, MicroChitosan NutriCology^®^, and Chitoseen™-F, which are marketed as fat reducers and cholesterol-reducing agents for obesity and weight management and Epakitin™, Nutri + Gen^®^, which are marketed as nutritional supplements and used to treat chronic kidney disease in dogs and cats. There are a large number of applications of chitosan in beverages, cosmetics, agriculture, and the paper industry; the biomedical applications of chitosan are mainly in the area of wound healing. Marketed wound dressings using chitosan include HemCon^®^ Bandage, TraumaStat^®^, ChitoGauze^®^ PRO, ChitoFlex^®^ PRO, Svek-Patch^®^, Chitodine^®^, Celox™, etc. ChitoSeat™ is a hemostatic sealant used as a surgical hemorrhage sealant for hard and soft tissues [174]. In addition to these formulations, there are various other chitosan-based formulations for drug or vaccine delivery currently under different stages of clinical investigations, as summarized in Table 5. The main challenge in the clinical applications of chitosan is the origin from natural sources; hence, its characterization and standardization are challenges. The structural characteristics of chitosan are dependent on the source of chitin, the extraction process, and the method and degree of deacetylation. Additionally, the formulation of chitosan into NPs introduces the need for additional controls related to nanoparticle size for long-term circulation and elimination [175]. Recently, chitosan has demonstrated immunostimulatory and adjuvant activity, and it is being explored as a delivery vehicle for vaccines, especially through the intranasal route [176,177]. However, in some cases, these immunostimulatory effects of chitosan can be undesirable if an excessive inflammatory response is induced. Although there are some obstacles involved in the clinical translation of chitosan NPs, there have been many formulations in different stages of clinical trials in the past 10 years. With various evolving standardization techniques, chitosan has the potential to become the delivery vehicle of choice for a number of clinical therapeutic applications.

## 8. Conclusions

Although chitosan is considered to be biodegradable and non-toxic and has been utilized in various marketed formulations in different countries for various dietary applications, the only FDA-approved pharmaceutical application of chitosan is for wound dressings. Chitosan has been the subject of a vast number of publications exploring its suitability in various forms for therapeutic drug delivery and for optimizing NP properties for drug loading and desired release profiles. However, the modifications of chitosan can alter its biological and toxicological profile, and we have many examples now of achieving a targeted and triggered release from chitosan-based NPs. Chitosan has been established as suitable for enhancing cellular uptake and improving bioavailability for various applications mainly due to its cationic nature and pH sensitivity; thus, chitosan provides an advantage for cellular targeting and controlled drug release at the site of action. The biological activity of chitosan in cells and in vivo is chiefly dependent on its size and charge and is boosted by its mucoadhesive properties. Longer-term toxicological studies of chitosan formulations for therapeutic applications need to be established for their successful clinical translation, particularly to ensure that intravenous chitosan formulations do not show evidence of rarely observed hematological toxicity, adverse effects upon pulmonary accumulation, or immune responses. These have not been commonly reported in animal models but should be considered. The versatility of chitosan to be modified physically and chemically to suit various applications provides a challenge for their regulatory approval, as the safety of all modifications and formulations needs to be considered on a case-by-case basis. Additionally, the immunological activation of chitosan and its ability to enhance penetration across the blood–brain barrier can prove to be undesirable in certain cases [4]. The origin of chitosan from natural sources further adds to unforeseeable challenges in their clinical translation. The standardization of extraction methods and analytical techniques for chitosan polymers would help enable faster translation to clinical applications. Despite the challenges, various formulations of chitosan are now in clinical trials. Chitosan nanoparticle formulations have great potential to achieve highly efficient, effective drug delivery by overcoming barriers to tissue and cellular uptake as well as drug release.

## Figures and Tables

**Table 1:** Properties of chitosan and its effect on cell interactions.

Properties of Chitosan	Cell Lines	Effect on Cell Interaction	Ref
Positive surface charge	A549, HKC, MRC-5, CCC-HSF-1, HUVEC, CRL-2472, UT-7, and K562	Promoted the internalization rate and increased the cellular uptake	[19]
	THP-1	Rapid internalization	[20]
	Caco-2	Rapid uptake in cells	[21]
	PC-3, PC12	Better adhesion of cell line to hyaluronic acid–chitosan films	[27]
pKa (6.5)	ARPE-19	Charge reversal and effective uptake	[10]
	SKOV-3, NCI/ADR-RES	pH-sensitive stealth coating and better association with cells	[24]
	HeLa, HaCaT, H1299 and NIH-3T3	Deprotonation at higher pH leads to desorption of fibronectin increasing detachment of cells	[26]
Hydrophilicity (deacetylation)/ Hydrophobicity (acetylation)	Fibroblast and chondrocytic cells	Higher acetylation resulting in increased hydrophobicity and lower surface charge leading to decreased adhesion of cells	[30]
	Olfactory epithelial cells	Higher degree of deacetylation beneficial for cell growth and adhesion	[30]
Permeability	Intestinal epithelium	Transiently open the epithelial tight junction by redistributing claudin-4 and facilitate penetration of insulin	[40,41,45]
	Caco-2	Dose-dependent transepithelial electrical resistance and increase permeability	[47]
	Calu-3	Displacement of zonula occludens-1 protein results in enhance permeability	[48]

**Table 2:** Various modifications of chitosan depicting different types of cellular uptake.

No.	Chitosan/Modifications	Ligands	Receptors	Cell Lines	Process of Uptake	Ref
1	Chitosan	Mannose	Mannose receptor	B16 melanoma tumor cells	Mannose receptor-mediated endocytosis	[69]
		Lactobionic acid bearing galactose	Asialoglycoprotein receptor	HeLa, CT-26, and Hep G2 cells	Asialoglycoprotein receptor-mediated endocytosis	[66]
		TAT-LHRH	LHRH receptor	BEL-7402 cells	LHRH receptor-mediated endocytosis	[67]
		Folic acid	Folate receptor	HeLa human cervical and SKOV3 ovarian cancer cells	Folate receptor-mediated endocytosis	[75]
		Folic acid	Folate receptor	HepG2 and HeLa cells	Folate receptor-mediated endocytosis	[72]
		Vitamin B12	Intrinsic factor receptor	Caco-2 cells	Passive diffusion and intrinsic factor receptor-mediated endocytosis	[76]
		Lauryl and succinyl moieties	Mucoadhesion	Caco-2 cells	Paracellular uptake	[77]
		Lactobionic acid	Asialoglycoprotein receptor	SMMC-7721 liver cancer cells	Asialoglycoprotein receptor-mediated endocytosis	[78]
		CD147 antibody	Asialoglycoprotein receptor	HepG2 liver cancer and SMMC-7721 cells	Caveolae-dependent pathway	[79]
2	Carboxy-methyl chitosan	HER-2/neu binding peptide	Human epidermal growth factor receptor 2	HEK 293 cells	Human epidermal growth factor receptor-mediated endocytosis	[11]
		Folic acid	Folate receptor	HepG2 cells	Folate receptor-mediated endocytosis	[80]
		Folic acid	Folate receptor	MCF-7 breast cancer cells	Folate receptor-mediated endocytosis	[81]
3	*N-*trimethyl chitosan	Galactose	Galactose receptors	QGY-7703 cells	Galactose receptor-mediated endocytosis	[68]
		Galactose	Asialoglycoprotein receptor	HepG2 human liver cancer cells	Galactose receptor-mediated endocytosis	[82]
		CSK peptide	HT29-MTX-E12 intestinal goblet cells	HT29-MTX-E12 cells	Clathrin- and caveolae-mediated endocytosis	[13]
4	PEGylated chitosan	EGFR targeting peptide	Asialoglycoprotein receptor	A549 human lung adeno carcinoma cells	Epidermal growth factor receptor-mediated endocytosis	[70]
5	GC-PDPA co-polymers	Estrogen	Estrogen receptor	MCF-7 cells	Estrogen receptor- mediated endocytosis	[71]
6	*N-*succinyl chitosan	ApoB100	LDL receptor	HepG2/ADM cells	Low density lipoprotein receptor-mediated endocytosis	[83]
7	*N-*succinyl-N -octyl chitosan	Folic acid	Folate receptor	Bel-7402 and A549 cells	Folate receptor-mediated endocytosis	[74]

**Table 3:** Significance of chitosan nanoparticles in prevention of premature elimination of loaded cargo.

No.	Causes of Premature Elimination	Chitosan/Modifications	Significance	Drug Loaded	Effect on Drug Distribution and Elimination	Ref
1	Non-specific uptake by spleen and liver	Chitosan	Positive surface charge	Cyclosporine	Lower apparent clearance and elimination rate constants; hence, longer circulation half-life and higher plasma AUC.	[130]
		Poly(methacrylic acid) functionalized chitosan	Negatively charged coating of PMAA	10-Hydroxy camptothecin	Significantly elongated blood circulation time from 12 to 24 h and reduced blood clearance (Cl) from 30.57 to 6.72 mL/h in vivo.	[87]
		Polyethylene glycol-conjugated chitosan oligosaccharide-arachidic acid	Stealth effect	Doxorubicin	Slower in vivo clearance rate subsequently extending the circulation time.	[131]
2	Opsonization	Chitosan funtionalized with poly(acrylic acid)	Colloidal stability and decreased protein adsorption capacity	None	Excellent stability in plasma and a remarkable buffering capacity.	[132]
3	Enzymatic degradation of biological drugs	O-carboxymethyl-chitosan/organosilica	Protection against DNase I and serum degradation	DNA complexes	Preventing pre-elimination of DNA and avoiding the dissociation of DNA in aqueous solution.	[133]
		Chitosan glutamate	Protection against enzymatic degradation	siRNA	Prevention of rapid degradation and better biological effect than naked siRNA.	[134]
4	Corneal clearance by metabolic enzymes	Methoxy poly(ethylene glycol)-poly(ε-caprolactone) and chitosan block polymer	Bioadhesion and prevents degradation	Diclofenac	Enhanced pre-corneal retention and penetration of the nanosuspension.	[135]
5	Physiological instability or aggregation	Glycol chitosan	Biocompatibility	Gold nanoparticles	Excellent stability and biocompatibility	[136]
6	Degradation by reactive oxygen species	Chitosan grafted with *N-*Acetyl-L-cysteine	Resistant to reactive oxygen species	Gold nanocluster	Reductant and stabilizer.	[137]

**Table 4:** Various modifications of chitosan depicting enhanced absorption and bioavailability.

No.	Chitosan Modification	Drug	Size (nm)	Zeta Potential (mV)	Targeting Site	Routes of Administration	References
1.	Polyethylene glycol-grafted chitosan	Insulin	150–300	+16 to +30	Mucosal absorption	Intranasal	[157]
2.	Carboxymethyl chitosan	Resveratrol	155.3 ± 15.2	10.28 ± 6.4	GIT	Oral	[158]
3.	Chitosan graft glyceryl mono-oleate	Enoxaparin	230.7 ± 7.3	21.6 ± 0.3	GIT	Intragastric	[159]
4.	*N-*trimethyl chitosan	Diclofenac Sodium	130–190	+4 to +9	Ocular	Ophthalmic	[160]
5.	PEGylated chitosan	Human parathyroid hormone 1-34	200–250	+35	Systemic circulation	Oral	[161]
6.	O-carboxymeymethy chitosan	Doxorubicin hydrochloride	250–300	−33.8 ± 1.6	pH responsive oral chemotherapy	Oral	[162]
7.	*N-*octyl-*N-*(2-carboxyl-cyclohexamethenyl) chitosan	Paclitaxel	145.9 ± 8.4	−14.8 ± 0.6	Tumor targeting	Intravenous	[163]
8.	Locus bean gum sulfate derivative-conjugated chitosan	Ovalbumin	180–200	+9 to +14	Immune reaction	Oral/Subcutaneous	[164]
9.	Cholesterol-modified glycol chitosan	Doxorubicin	237–336	--	Tumor targeting	Intravenous administration	[165]

**Table 5:** Chitosan-based formulations currently in clinical trials (Source: Clinical trials.gov identifier, accessed on 21 March 2020).

No.	Clinical Trial Phase	Year	Composition	Type of Formulation
1	Phase 1	2010	Chitosan + mannitol + sucrose + monophosphoryl lipid adjuvant	Intranasal vaccine
2	Phase 1b/2	2021	Chitosan	Oral supplement
3	Phase 2/3	2019	Chitosan NPs	Oral irrigation solution
4	Phase 4	2014	Chitosan	Solution (12 mg/mL)
5	Not listed	2018	Chitosan nanoparticle gel	Oral irrigation solution
6	Phase 1	2011 (recently published in 2019)	Chitosan-*N*-acetylcysteine (Lacrimera^®^)	Eye drops
7	Phase 2	2007	HEP-40 chitosan (enzymatic polychitosamine hydrolysate) (Libracol^®^)	Oral
8	Phase 3	2016	Chitosan + isosorbide dinitrate versus either alone	Gel spray
9	Phase 3	2017	Chitosan + ketamine	Intranasal spray
